# The revision of chromosome III (EF) mapping in *Chironomus plumosus* (Linnaeus, 1758) group (Diptera, Chironomidae)

**DOI:** 10.3897/CompCytogen.v12i2.23327

**Published:** 2018-06-06

**Authors:** Veronika V. Golygina, Iya I. Kiknadze

**Affiliations:** 1 Institute of Cytology and Genetics SB RAS, Prosp. akademika Lavrentieva 10, Novosibirsk 630090, Russia; 2 Novosibirsk State University, ul. Pirogova, 2, Novosibirsk, 630090, Russia

**Keywords:** *Chironomus plumosus*, *Chironomus*, Chironomidae, karyotype, polytene chromosome, banding sequence, chromosome III (EF), chromosome mapping, phylogeny, phylogenetic relationship, karyological analysis

## Abstract

A revision of mapping of main and alternative banding sequences in chromosome III (EF) has been made for 14 species of the *Chironomus plumosus* group. In total, new versions of mapping are presented for 18 banding sequences of arm E and 18 banding sequences of arm F. A new way of tracing the origins of banding sequences in chromosome III of the *Ch. plumosus* group in comparison with basic banding sequences of the genus *Chironomus* is suggested. The presented data indicate that h’pluE2 in arm E and p’borF2 in arm F are the closest to banding sequences of *Ch. piger* Strenzke, 1959 and thus should be considered the most ancient among banding sequences of chromosome III in the *Ch. plumosus* group. Phylogenetic relationships of banding sequences of chromosome III are discussed.

## Introduction

The *Chironomus plumosus* group of sibling species presents a great opportunity for the study of the genomic reorganization at the chromosome level during speciation as most of the sibling species have wide geographic ranges with high levels of chromosomal polymorphism in natural populations (Kiknadze 1987, Kiknadze et al. 1987, 2000, Shobanov 1994b, Michailova and Petrova 1991, Petrova et al. 1996, Golygina et al. 1996, Butler et al. 1999, Golygina 1999, Gunderina et al. 1999, Golygina and Kiknadze 2001, Golygina et al. 2007). The possibility of mapping all the karyotypes in the genus *Chironomus* Meigen 1803 against one standard species allows us to detect all chromosomal rearrangements that distinguish different species and reconstruct their phylogenetic relationship on the basis of karyological analysis (Keyl 1962, Wülker et al. 1989, Shobanov and Zotov 2001, Kiknadze et al. 2004b, 2008, 2016, Gunderina et al. 2005a). However, for conducting such studies it is very important to have high-resolution photographic maps of karyotypes and a unified mapping system of polytene chromosomes. In our earlier works (Golygina and Kiknadze 2008, 2012) we extensively discussed the general difficulties facing a researcher who works with the *Ch. plumosus* group and presented a revision of mapping for chromosome I (AB) and II (CD). However, the situation with mapping of banding sequences in chromosome III (EF) has an additional problem.

Arm E is the most conservative arm in karyotypes of *Ch. plumosus* sibling species, as well as in the genus *Chironomus* (Keyl 1962, Wülker 1989, Kiknadze et al. 2004a, Gunderina et al. 2005b, Golygina et al. 2007). Despite the fact that the established relationships between banding sequences of *Ch. plumosus* sibling species in this arm are quite simple, the situation with mapping is rather complicated due to the presence of two versions of mapping of banding sequence h’pluE1 in comparison with h’pigE1 – the standard banding sequence of *Ch. piger* Strenzke, 1959. The first version of h’pluE1 mapping was presented by Keyl (1962). This version of h’pluE1 mapping was used until 1999 by all authors who worked with the Keyl mapping system and h’pluE1 was considered the closest to h’pigE1 among banding sequences of arm E in *Ch. plumosus* group. At the same time in most works on inversion polymorphism in populations of species from *Ch. plumosus* group the Maximova mapping system (Maximova 1976, Shobanov 1994a), designed for mapping of chromosomes only in this group, was used. Because of this no other banding sequences found in *Ch. plumosus* sibling species were actually directly compared to h’pigE1 but rather mapped using h’pluE1 as a reference. In 1999 we performed an extensive analysis of banding sequences h’pluE1 and h’pluE2 and suggested that the true relationships between h’pigE1, h’pluE1 and h’pluE2 are different from those assumed previously (Golygina 1999, Butler et al. 1999, Golygina and Kiknadze 2001). For example, the comparison of h’pluE2 to h’pigE1 and banding sequences in arm E of other *Chironomus* species indicated that h’pluE2 is actually identical to the banding sequence considered basic for the genus, which is present in karyotypes of several species, such as *Ch. acidophilus* Keyl, 1960, *Ch. luridus* Strenzke, 1959, *Ch. yoshimatsui* Martin & Sublette, 1972 etc. (Wülker 1980). This banding sequence differs from h’pigE1 by single inversion and thus h’pluE2 is closer to h’pigE1 than h’pluE1. Moreover, a revision of h’pluE1 breakpoints was suggested (Golygina 1999, Butler et al. 1999), which means that virtually all banding sequences in arm E of other species from *Ch. plumosus* group required a revision as they are either identical to or originating from the h’pluE1.

However, h’pluE1 is also considered to be identical to banding sequences in arm E of many species from the genus *Chironomus* Meigen outside the *Ch. plumosus* group, such as *Ch. aberratus* Keyl, 1961, *Ch. anthracinus* Zetterstedt, 1860, *Ch. cucini* Webb, 1969, *Ch. jonmartini* Lindeberg, 1979 and several others (Keyl 1962, Wülker 1980, Kiknadze et al. 2004a, 2016). Thus, in papers discussing evolution of banding sequences in the genus *Chironomus*, including other *Ch. plumosus* sibling species, Keyl’s original version of the mapping of h’pluE1 has been used (Kiknadze et al. 2004a). This situation makes comparison of data from different papers increasingly difficult so complete revision of banding sequences from the arm E in *Ch. plumosus* group was required.

Banding sequences of arm F also show a high level of conservatism among *Chironomus* species, although it is not as high as in arm E (Keyl 1962, Wülker 1980, Kiknadze et al. 2004a, 2016). The first mapping of arm F of *Ch. plumosus* was published by Keyl (1962) for banding sequence h’pluF1 and this version was used by all authors until now. Yet our analysis indicated that changes to this mapping should be made and thus, as for the arm E, banding sequences of several species in the group required a revision.

In this paper we present the results of revision of mapping for main (present in homozygotes in most populations with high frequencies) and alternative (present in homozygotes in some populations with high frequencies and in heterozygotes in most populations) banding sequences in chromosome III (EF) of 14 sibling species belonging to *Ch. plumosus* group.

## Material and methods

Revision of chromosome III (EF) mapping was conducted for 14 *Ch. plumosus* sibling species: *Chironomus agilis* Shobanov & Djomin, 1988, Ch. sp. prope agilis (working name “*Ch. agilis* 2”) (Kiknadze et al. 1991a), *Ch. balatonicus* Dévai, Wülker & Scholl, 1983, *Ch. bonus* Shilova & Dzhvarsheishvili, 1974, *Ch. borokensis* Kerkis, Filippova, Shobanov, Gunderina & Kiknadze, 1988, *Ch. entis* Shobanov, 1989, *Ch. muratensis* Ryser, Scholl & Wülker, 1983, *Ch. nudiventris* Ryser, Scholl & Wülker, 1983, *Ch. plumosus* (Linnaeus, 1758), *Ch. sinicus* Kiknadze, Wang, Istomina & Gunderina, 2005, *Chironomus* sp. J (Kiknadze et al. 1991b), *Chironomus* sp. K (Golygina and Ueno 2005), *Ch. suwai* Golygina & Martin, 2003, *Ch. usenicus* Loginova & Belyanina, 1994. High-resolution photomaps of all banding sequences created from chromosome slides prepared from the salivary glands of 4^th^ instar larvae by standard aceto-orcein method (Kiknadze et al. 1991b).

Mapping of arms E and F was done according to Keyl-Devai mapping system (Keyl 1962, Dévai et al. 1989) with *Ch. piger* chromosomes as the standard.

Each banding sequence in each chromosomal arm is given a short designation as follows: three-letter abbreviation of the species name (for example, agi – for *Ch. agilis*, bal – for *Ch. balatonicus* etc.) is followed by the name of the arm and the serial number of banding sequence in this arm (according to the order of its discovery), and prefixed by a letter that indicates its geographical distribution – p’ for Palearctic sequences, n’ for Nearctic sequences, or h’ for Holarctic sequences (e.g. p’balE1, h’pluE2, n’entF4 etc.).

Equipment of the Centre of Microscopical analysis of biological objects SB RAS in the Institute of Cytology and Genetics (Novosibirsk) was used for this work: microscope “Axioskop” 2 Plus, CCD-camera AxioCam HRc, software package AxioVision 4 (Zeiss, Germany).

## Results

### Arm E

As was mentioned above, two versions of mapping of banding sequence h’pluE1 in comparison with standard banding sequence h’pigE1 are used in different publications.

The Keyl version suggests two inversion steps between h’pluE1 and h’pigE1 as follows (Keyl 1962): 
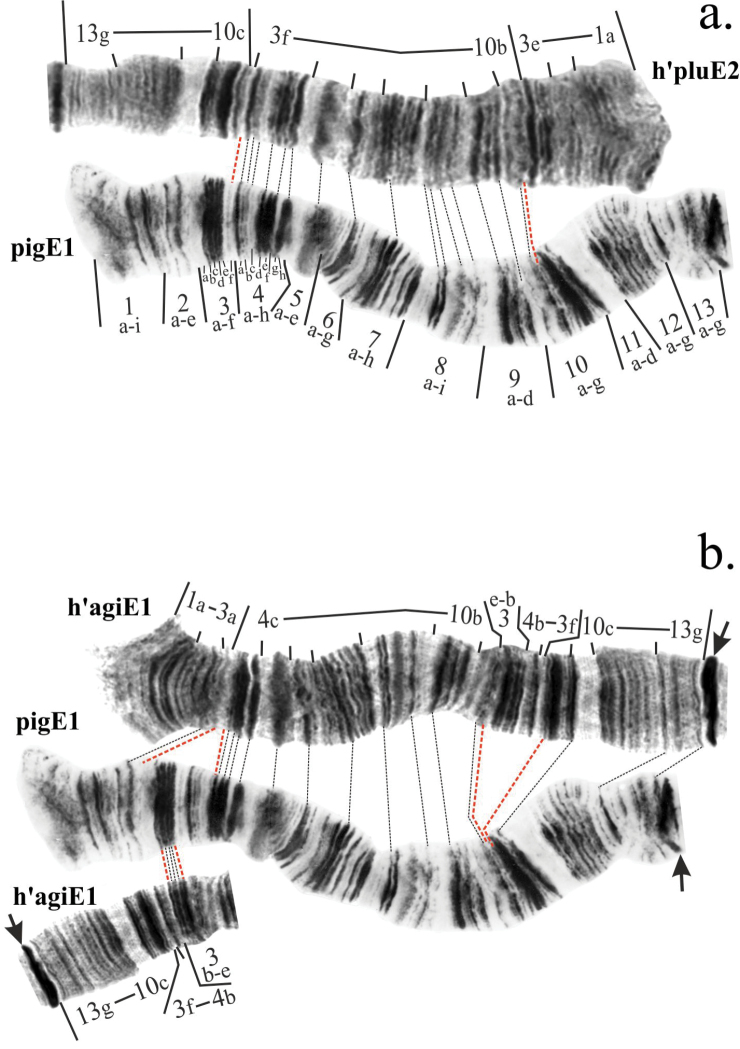


This hypothetical banding sequence has never been found in any studied karyotypes of *Chironomus* species.

**Figure 1.**
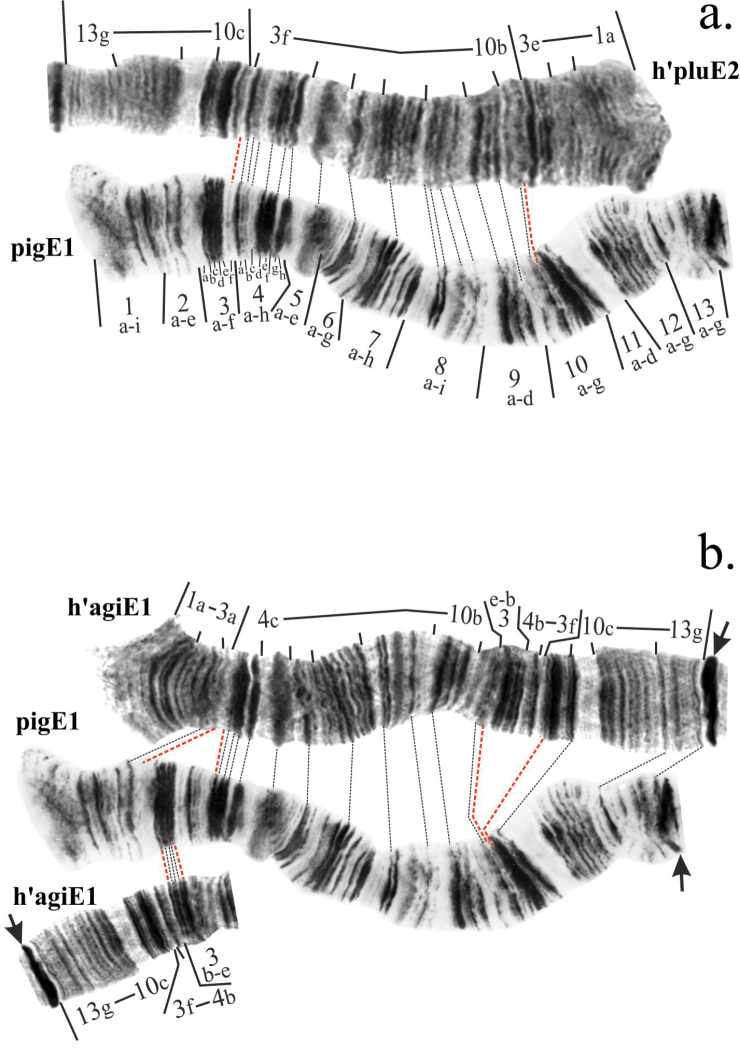
Mapping comparison of banding sequences h’pigE1, h’pluE2 and h’agiA1 (identical to h’pluE1). a – comparison of h’pigE1 and h’pluE2, b – comparison of h’pigE1 and h’agiE1=h’pluE1. Centromeric bands are designated by arrows. Individual band in regions 3 and 4 of h’pigE1 are marked by small letters. Dotted lines connect identical discs in compared banding sequences. Red dotted lines indicate borders of regions, where banding patterns of compared banding sequences are identical.

As h’pluE2 differs from h’pluE1 by simple inversion but initially was not directly compared to h’pigE1, its previous mapping was a derivative from h’pluE1 mapping (Table 1) and placed it within 3 inversion steps from h’pigE1: 
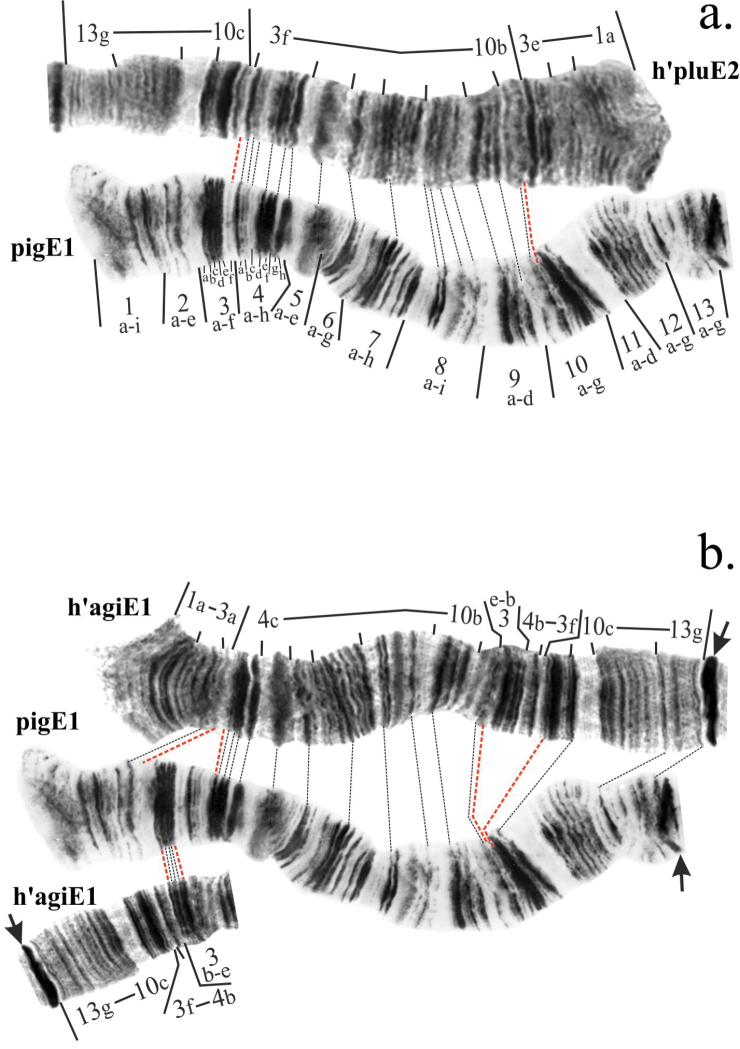


However, our study of these three banding sequences leads us to believe that h’pluE2 is in fact closer to h’pigE1 and h’pluE1 originated from it, which required a slightly different position of inversion breakpoints: 
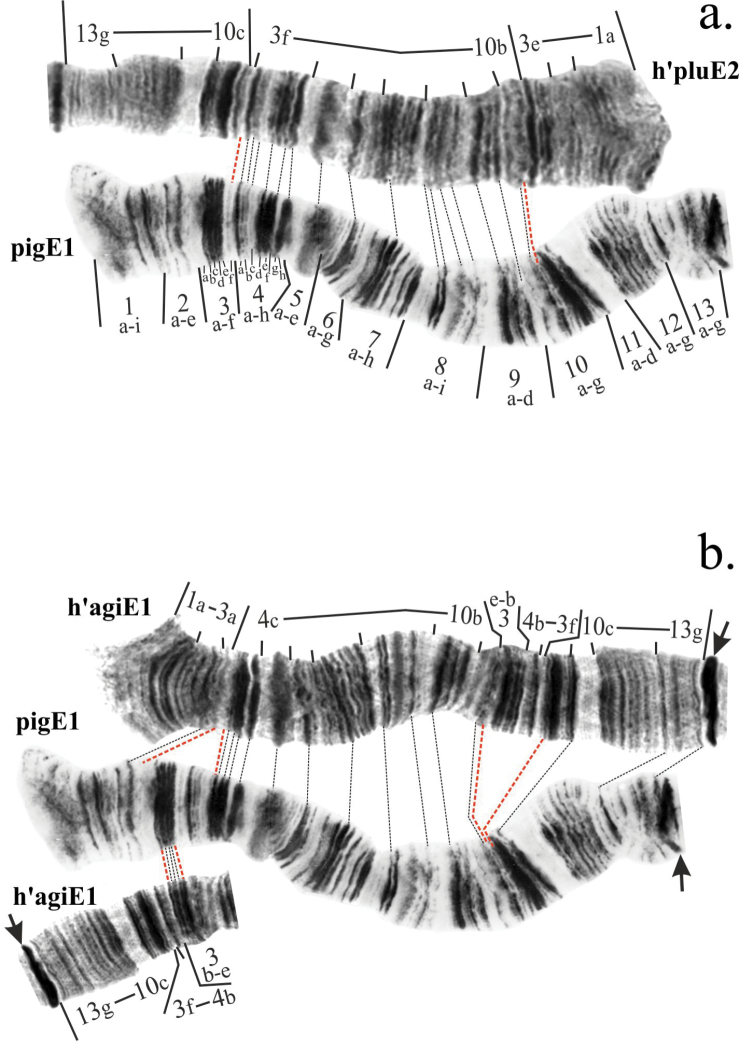


Reasons for the suggested change in mapping of h’pluE1 and h’pluE2 are shown on Figure 1a, b where comparison of regions 3 and 4 of arm E of *Ch. plumosus*, *Ch. agilis* and *Ch. piger* are presented.

**Table 1.** Mapping of arm E main and alternative banding sequences in *Ch. plumosus* group before the revision.

Designation of banding sequence	Mapping of banding sequence
h’agiE1*^†^	=h’pluE1 KV^‡^: (Shobanov and Djomin 1988, Kerkis et al. 1989a, Kiknadze et al. 1991b, 1996b, 2004a, Shobanov and Zotov 2001, Michailova et al. 2002) ^§^
h’agi2E1*	=h’pluE1 KV: (Kiknadze et al. 1991a, 2004a)
p’balE1*	=h’pluE1 KV: (Dévai et al. 1983, Kiknadze and Kerkis 1986, Kiknadze 1987, Kiknadze et al. 1991b)
h’bonE1*	=h’pluE1 KV: (Kerkis et al. 1989, Kiknadze et al. 1991b, 2004a, Shobanov and Zotov 2001)
h’borE1*	=h’pluE1 KV: (Kerkis et al. 1988, 1989a, Kiknadze et al. 1991b, 1996b, 2004a, Shobanov and Zotov 2001)
h’entE1*	KV: 1a-2e 10g-10c 3f-4h 10b-5a 3e-a 11a-13g C (Golygina 1999, Kiknadze et al. 2000, 2004a, Proviz and Bazova 2013) **^|^** GV: 1a-2e 10g-10c 3f-4b 3b-e 10b-4c 3a 11a-13g C (Golygina 1999, Kiknadze et al. 2000)
h’entE2	=h’pluE1 KV: (Dyomin and Shobanov 1990, Golygina 1999, Kiknadze et al. 2000, Proviz and Bazova 2013) GV: (Golygina 1999, Kiknadze et al. 2000)
h’murE1*	=h’entE1^¶^ KV version 1: 1a-3e 4a-h 10b-5a 11d-10c 3f 12a-13g C (Ryser et al. 1983, Kiknadze and Kerkis 1986, Kiknadze 1987, Wülker et al. 1989) KV version 2: 1a-2e 10g-10c 3f-4h 10b-5a 3e-a 11a-13g C (Kiknadze et al. 2004a)
h’nudE1*	=h’pluE1 KV: (Ryser et al. 1983, Kiknadze et al. 1987, 1991b, 2004a)
h’nudE2	=h’murE1^#^ KV: 1a-3e 4h-a 10b-5a 11d-10c 3f 12a-13g C (Kiknadze et al. 1987)
h’pluE1*	KV: 1a-3e 5a-10b 4h-3f 10c-13g C (Keyl 1962, Kiknadze 1987, Wülker et al. 1989, Kiknadze et al. 1991b, 1996b, 2004, Butler et al. 1999, Golygina 1999, Golygina and Kiknadze 2001, Michailova et al. 2002) GV: 1a-3a 4c-10b 3e-b 4b-3f 10c-13g C (Butler et al. 1999, Golygina 1999, Golygina and Kiknadze 2001)
h’pluE2	KV: 1a-3a 4d-h 10b-3b 4c-3f 10c-13g C (Butler et al. 1999, Golygina 1999, Golygina and Kiknadze 2001) GV: 1a-3e 10b-3f 10c-13g C (Butler et al. 1999, Golygina 1999, Golygina and Kiknadze 2001)
h’sinE1*	=h’pluE1 KV: (Kiknadze et al. 2005) GV: (Kiknadze et al. 2005)
h’spJE1*	=h’pluE1 KV: (Kiknadze et al. 2004a)
h’spKE1*	=h’pluE1 GV: (Golygina and Ueno 2008)
h’suwE1*	=h’pluE1 KV: (Golygina et al. 2003, Kiknadze et al. 2004a) GV: (Golygina et al. 2003)
p’useE1*	KV: 1a-3e 5a 3f-4h 10b-5b 10c-13g C (Loginova and Belyanina 1994)
h’useE3	=h’pluE1 KV: (Loginova and Belyanina 1994)

^†^ – main banding sequences are marked by *, ^‡^ – KV – variant of mapping done according to Keyl’s version of mapping of banding sequence h’pluE1 (Keyl 1962), GV – variant of mapping done according to Golygina’s version of mapping of banding sequence h’pluE1 (Golygina 1999) ^§^– papers with given version of the mapping are shown in parenthesis, **^|^** – shown only the last version of mapping of this banding sequence as there were several other papers published earlier – Kerkis et al. 1989, Dyomin and Shobanov 1990, Kiknadze et al. 1991c – with different mapping variants, ^¶^ – the fact that h’murE1 is identical to h’entE1 was not known until 2004 so these banding sequences were mapped separately in earlier works and their mapping differed from one another, ^#^– while h’nudE2 is identical to h’murE1 and h’entE1 the mapping of h’nudE2 published by Kiknadze and coauthors in 1987 differs from the mapping published later for h’murE1 (Kiknadze et al. 2004).

As all banding sequences in arm E of other species from *Ch. plumosus* group are either identical to or originated from the h’pluE1 it was required to make a revision of all of them.

**Figure 2.**
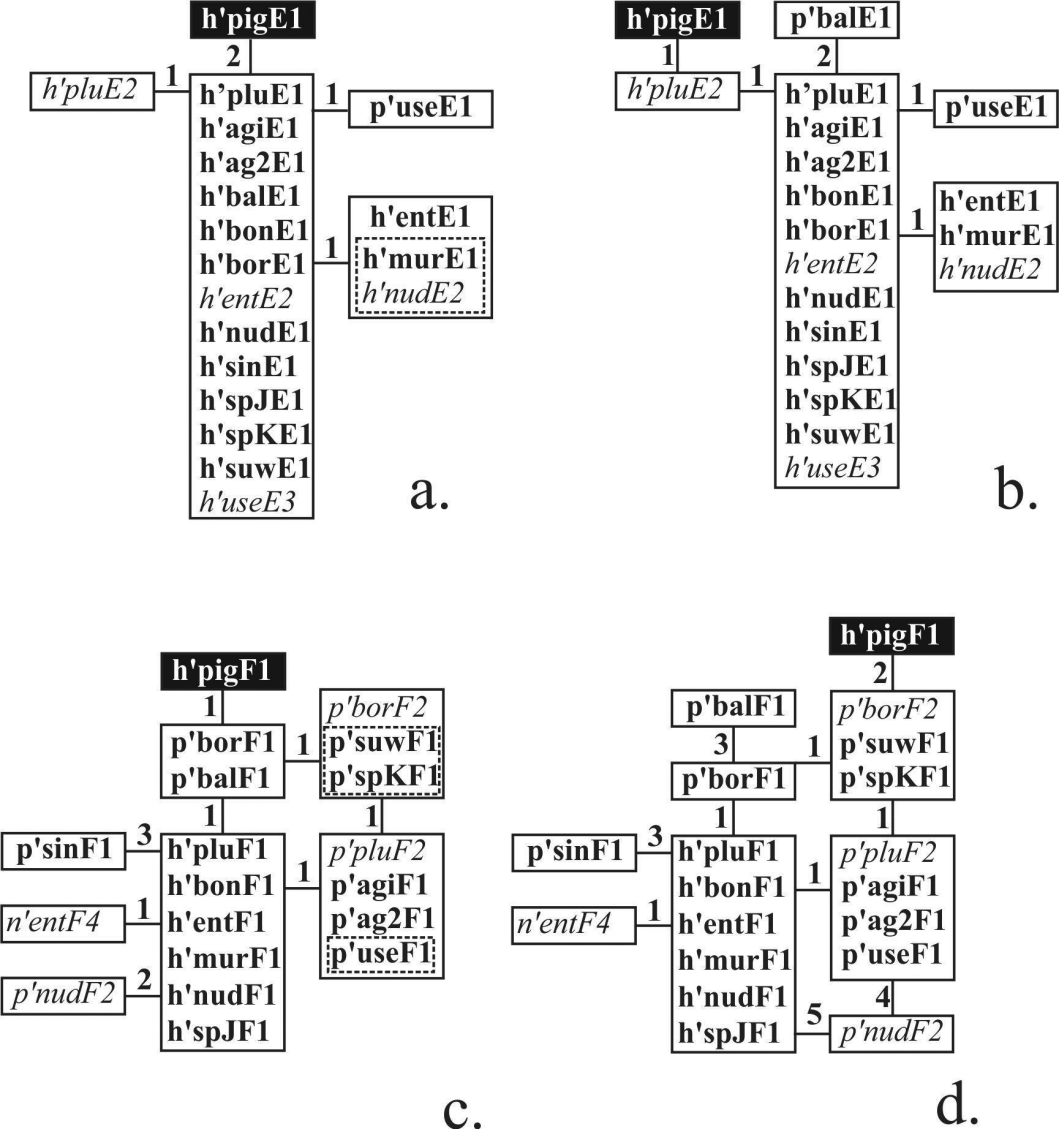
Phylogenetic relationship of main and alternative banding sequences in arms E and F before (**a, c**) and after (**b, d**) the revision. Main banding sequences are written in bold, alternative – in italic. Identical banding sequences enclosed in boxes, figures near the lines that connect banding sequences indicate numbers of inversion steps between them. Dotted lines enclosing some banding sequences inside a block indicate that mapping presented for these banding sequences differ from mappings of other banding sequences in the block, yet all banding sequences in the block were considered identical.

Mapping of banding sequences according to the Keyl-Devai system for *Ch. plumosus* sibling species published up to now, is shown for both versions in Table 1. In total 18 banding sequences (14 main and 4 alternative) are considered in this study. A dendrogram of banding sequences constructed on the basis of published mapping using Keyl’s version of h’pluE1 is shown in Fig. 2a, where main banding sequences are written in bold and alternative banding sequences in italics. As can be seen, 12 banding sequences were considered to be identical to h’pluE1 with five other banding sequences originating from h’pluE1 by one simple inversion (three of which – h’entE1, h’murE1 and h’nudE2 – were considered identical to each other).

**Figure 3.**
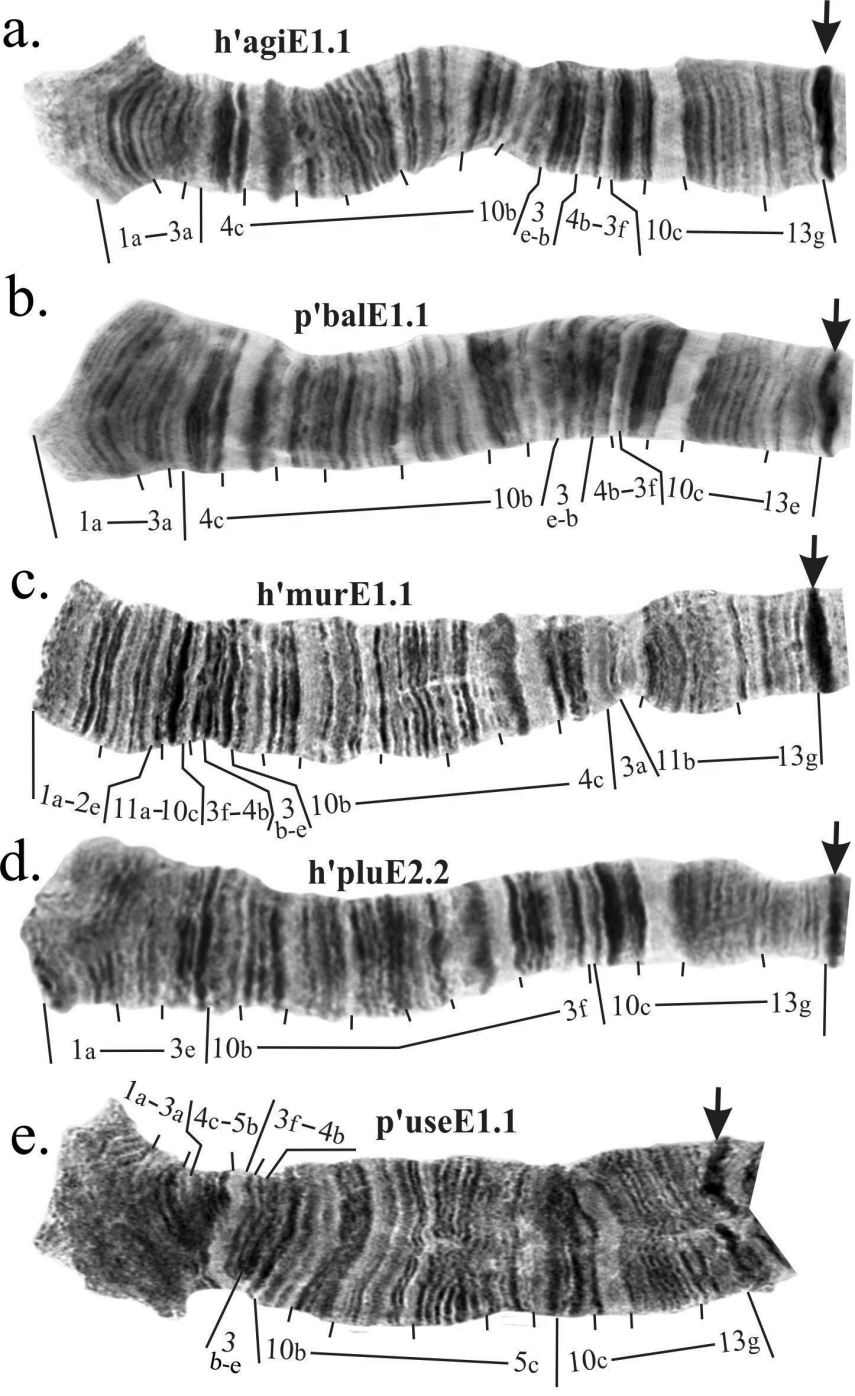
Mapping of banding sequences of *Ch. plumosus* sibling species in arm E after the revision. **a** h’agiE1.1 (identical to h’pluE1, h’ag2E1, h’bonE1, h’borE1, h’entE2, h’nudE1, h’sinE1, h’spJE1, h’spKE1, h’suwE1, h’useE3) **b** p’balE1.1 **c** h’murE1.1 (identical to h’entE1, h’nudE2) **d** h’pluE2.2 **e** p’useE1.1. Centromeric bands are designated by arrows.

According to our analysis, the true phylogenetic relationships are shown on Figure 2b. Eleven banding sequences are indeed identical to h’pluE1 so the changes in their mapping had to be made in accordance with h’pluE1 mapping (Table 2, Fig. 3a). Four banding sequences – h’entE1, h’murE1, h’nudE2 and p’useE1 – required minor corrections of inversion breakpoints, which differentiate them from h’pluE1. One banding sequence – p’balE1 – required a major revision.

### The revision of arm E mapping of *Ch. balatonicus*

**Figure 4.**
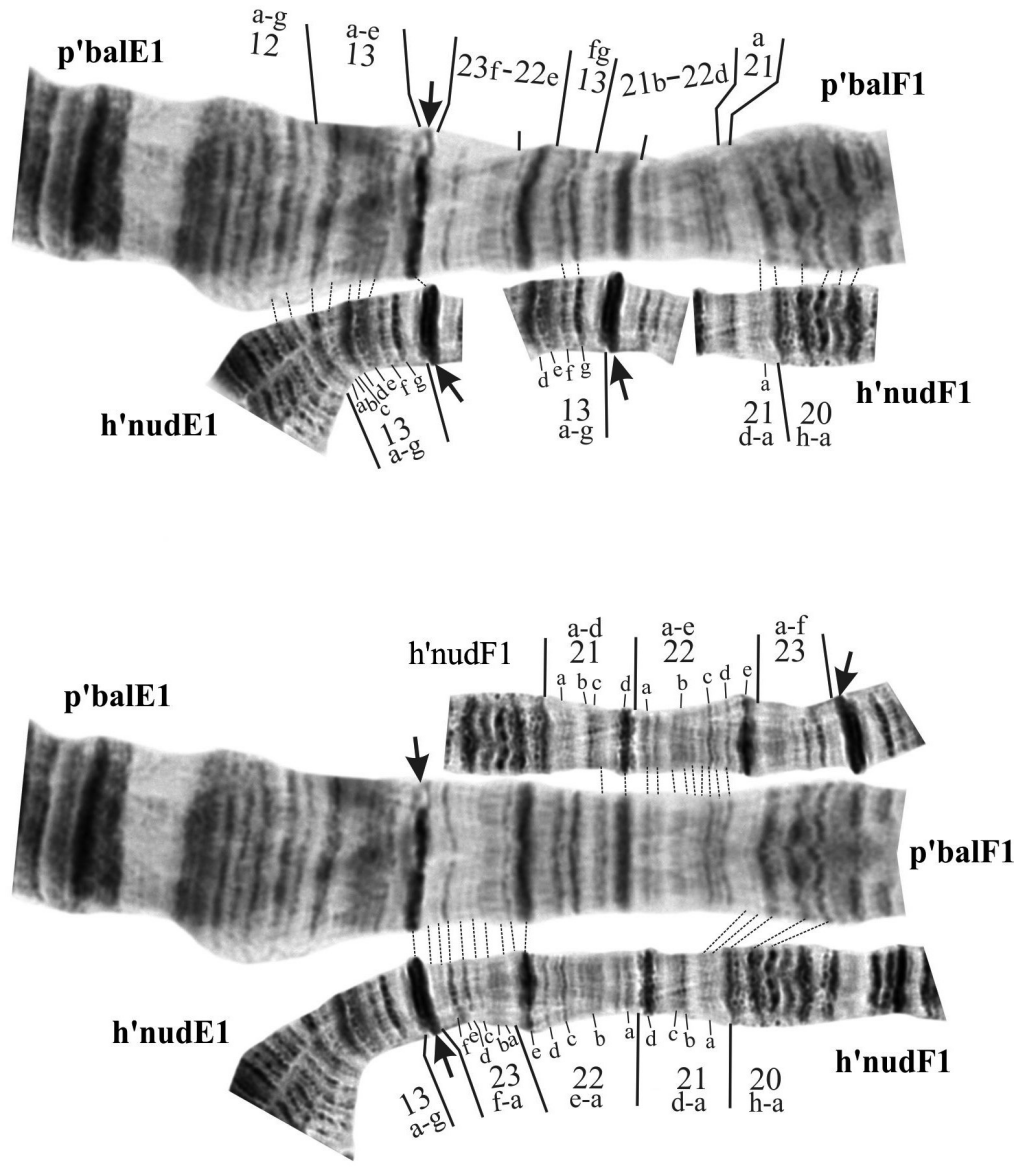
Mapping of pericentric inversion on chromosome EF of *Ch. balatonicus*. Individual bands in regions are marked by small letters.

It was believed previously that the main banding sequence of *Ch. balatonicus* is identical to h’pluE1. However, our analysis had shown that this species differs from all other species of *Ch. plumosus* group by the presence of complex pericentric inversion in chromosome EF (Figs 3b, 4). As a result of this inversion bands 13fg transferred from arm E into arm F of *Ch. balatonicus* so while on the most length of the arm banding pattern of p’balE1 is indeed identical with h’pluE1, *Ch. balatonicus* arm E is shorter than arm E of the rest of *Ch. plumosus* group species by 2 bands (Table 2). The more detailed analysis of this inversion is presented in revision of arm F below (Fig. 4).

### The revision of arm E mapping of *Ch. entis*, *Ch. muratensis*, and *Ch. nudiventris*

Banding sequences h’entE1, h’murE1, and h’nudE2 are identical and differ from h’pluE1 by one simple paracentric inversion. We believe that minor revision should be made for its breakpoints (Fig. 3c, Table 2).

### The revision of arm E mapping of *Ch. usenicus*

Banding sequence p’useE1 differs from h’pluE1 by simple inversion. Loginova and coauthors (Loginova et al. 1994) placed the left inversion breakpoint between bands 5a and 5b but closer analysis shows that the real breakpoint is situated between bands 5b and 5c as the latter – the wide fuzzy dark band – closely adjoins region 10c-g (Fig. 3e, Table 2).

**Table 2.** Mapping of arm E main and alternative banding sequences in *Ch. plumosus* group after the revision.

**Designation of banding sequence**	**Mapping of banding sequence**
h’agiE1*^†^	=h’pluE1
h’agi2E1*	=h’pluE1
p’balE1*	KV: 1a-3e 5a-10b 4h-3f 10c-**13e** C ^‡^ GV: 1a-3a 4c-10b 3e-b 4b-3f 10c-**13e** C^§^
h’bonE1*	=h’pluE1
h’borE1*	=h’pluE1
h’entE1*	KV: KV: 1a-2e **11a**-10c 3f-4h 10b-5a 3e-a **11b**-13g C GV: 1a-**2e 11a**-10c 3f-4b 3b-e 10b-4c **3a 11b**-13g C
h’entE2	=h’pluE1
h’murE1*	=h’entE1
h’nudE1*	=h’pluE1
h’nudE2	=h’entE1
h’pluE1*	KV: 1a-3e 5a-10b 4h-3f 10c-13g C GV: 1a-3a 4c-10b 3e-b 4b-3f 10c-13g C
h’pluE2	KV: 1a-3a 4d-h 10b-3b 4c-3f 10c-13g C GV: 1a-3e 10b-3f 10c-13g C
h’sinE1*	=h’pluE1
h’spJE1*	=h’pluE1
h’spKE1*	=h’pluE1
h’suwE1*	=h’pluE1
p’useE1*	KV: 1a-3e 5ab 3f-4h 10b-5c 10c-13g C GV: 1a-3a 4c-**5b** 3f-4b 3b-e 10b-**5c** 10c-13g C
h’useE3	=h’pluE1

^†^ – main banding sequences are marked by *, ^‡^ – KV – variant of mapping done according to Keyl’s version of mapping of banding sequence h’pluE1 (Keyl 1962), GV – variant of mapping done according to Golygina’s version of mapping of banding sequence h’pluE1 (Golygina 1999), ^§^ – parts of the sequences highlighted in bold indicate regions which mapping had been changed as a result of the revision.

### Arm F

Banding patterns in arm F of *Chironomus* species are not as conservative as in arm E, but the arm is still considered to have a low level of polymorphism with many species sharing the same banding sequences and a lot of species that differ from each other by single inversion steps (Keyl 1962, Wülker 1989, Kiknadze et al. 2004a, Gunderina et al. 2005b, Golygina et al. 2007). *Ch. plumosus* was the first species in the *Ch. plumosus* group which arm F’s banding sequence was mapped so it became the template to map all other species in the group. Mapping of h’pluF1 was first presented by Keyl (1962) and according to it h’pluF1 differs from h’pigF1 by two non-overlapping paracentric inversions – one in the region 1e-6e and the other in the region 11a-17d. According to this version of mapping region 10 remain unbroken. When karyotype of *Ch. borokensis* was described (Kerkis at al. 1988) the mapping of p’borF1 presented in the paper placed it as an intermediate banding sequence between h’pigF1 and h’pluF1, which differs from h’pigF1 by single inversion in the region 11a-17d (Table 3). Later when banding sequences of other species in the group were described, their mapping was based on these two assumptions so in all of them region 10 was mapped as whole. Thus basically all main banding sequences of *Ch. plumosus* group species differ from each other by combination of presence or absence of inversions in regions 1e-6e and 11a-17d (Table 3). The relationships of banding sequences in arm F as they were presumed to be up until now are shown on Figure 2c.

However, our analysis had clearly shown that the inversion that was previously defined as 11a-17d actually has different breakpoints, which in turn required re-evaluating relationships both between banding sequences inside the *Ch. plumosus* group and with the standard h’pigF1.

**Table 3.** Mapping of arm F main and alternative banding sequences in *Ch. plumosus* group before the revision.

Designation of banding sequence	Mapping of banding sequence
p’agiF1*^†^	=p’pluF2 While all authors considered it to be identical to p’pluF2, the presented mapping of the banding sequence was different in different papers: 1a-d 6e-1e 7a-10d 18c-a 11a-17d 18d-23f C (Shobanov and Djomin 1988, Shobanov and Zotov 2001, Kiknadze et al. 2004a) ^‡^ 1a-d 6e-1e 7a-10d 18e-a 11a-17d 19a-23f C (Kerkis et al. 1989a, b, Kiknadze et al. 1991b?, 1996b, Michailova et al. 2002)
p’agi2F1	=p’agiF1 While authors stated that it is identical to p’agiF1, the presented mapping of the banding sequence was different in different papers: 1a-d 6e-1e 7a-10d 17d-a 11a-16g 18a-23f C (Kiknadze et al. 1991a) 1a-d 6e-1e 7a-10d 18c-a 11a-17d 18d-23f C (Kiknadze et al. 2004a)
p’balF1	=h’borF1 (Dévai et al. 1983, Kiknadze and Kerkis 1986, Kiknadze 1987, Kiknadze et al. 1991b, Michailova and Krastanov 2000, Michailova et al. 2002)
p’bonF1	=h’pluF1 (Kerkis et al. 1989b, Kiknadze et al. 1991b, 2004, Shobanov and Zotov 2001)
p’borF1	1a-10d 17d-11a 18a-23f C (Kerkis et al. 1988, 1989a, Kiknadze et al. 1991b?, 1996a, 2004a)
p’borF2	no published mapping according to Keyl-Devai system
h’entF1	=h’pluF1 (Kerkis et al. 1989a, b, Dyomin and Shobanov 1990, Golygina 1999, Kiknadze et al. 2000, 2004a, Proviz and Bazova 2013, Shobanov and Zotov 2001)
n’entF4	1a-d 6e-1e 19d-18a 11a-17d 10d-7a 20a-23f C (Kiknadze et al. 2000)
h’murF1	=h’pluF1 (Ryser et al. 1983, Kiknadze and Kerkis 1986, Kiknadze 1987, Kiknadze et al. 2004a)
h’nudF1	=h’pluF1 (Ryser et al. 1983, Kiknadze et al. 1987, 1991b, 2004a)
p’nudF2	1a-d 14a-15i 19d-18a 11a-13d 6e-1e 7a-10d 17d-16a 20a-23f C (Kiknadze et al. 1987)
h’pluF1	1a-d 6e-1e 7a-10d 17d-11a 18a-23f C (Keyl 1962, Kiknadze 1987, Wülker et al. 1989, Kiknadze et al. 1991b, 1996b, Butler et al. 1999, Golygina 1999, Michailova and Krastanov 2000, Golygina and Kiknadze 2001, Michailova et al. 2002, Kiknadze et al. 2004a, Proviz and Bazova 2013)
p’pluF2	1a-d 6e-1e 7a-10d 18c-a 11a-17d 18d-23f C (Butler et al. 1999) 1a-d 6e-1e 7a-10b 18e-a 11a-17d 10dc 19a-23f C (Golygina 1999, Golygina and Kiknadze 2001)
p’sinF1	1a-d 6e-5d 10d-7a 5c-1e 14f-17d 14e-11a 18a-23f C (Kiknadze et al. 2005)
h’spJF1	=h’pluF1 (Kiknadze et al. 1991b)
p’spKF1	=p’suwF1 (Golygina and Ueno 2008)
p’suwF1	=p’borF2 1a-10b 18e-a 11a-17d 10dc 19a-23f C (Golygina et al. 2003, Kiknadze et al. 2004a)
p’useF1	1a-d 6e-1e 7a-10d 18e-a 11a-17d 19a-23f C (Loginova and Beljanina 1994)

^†^ – main banding sequences are marked by *, ^‡^ – papers with given version of the mapping are shown in parenthesis.

**Figure 5.**
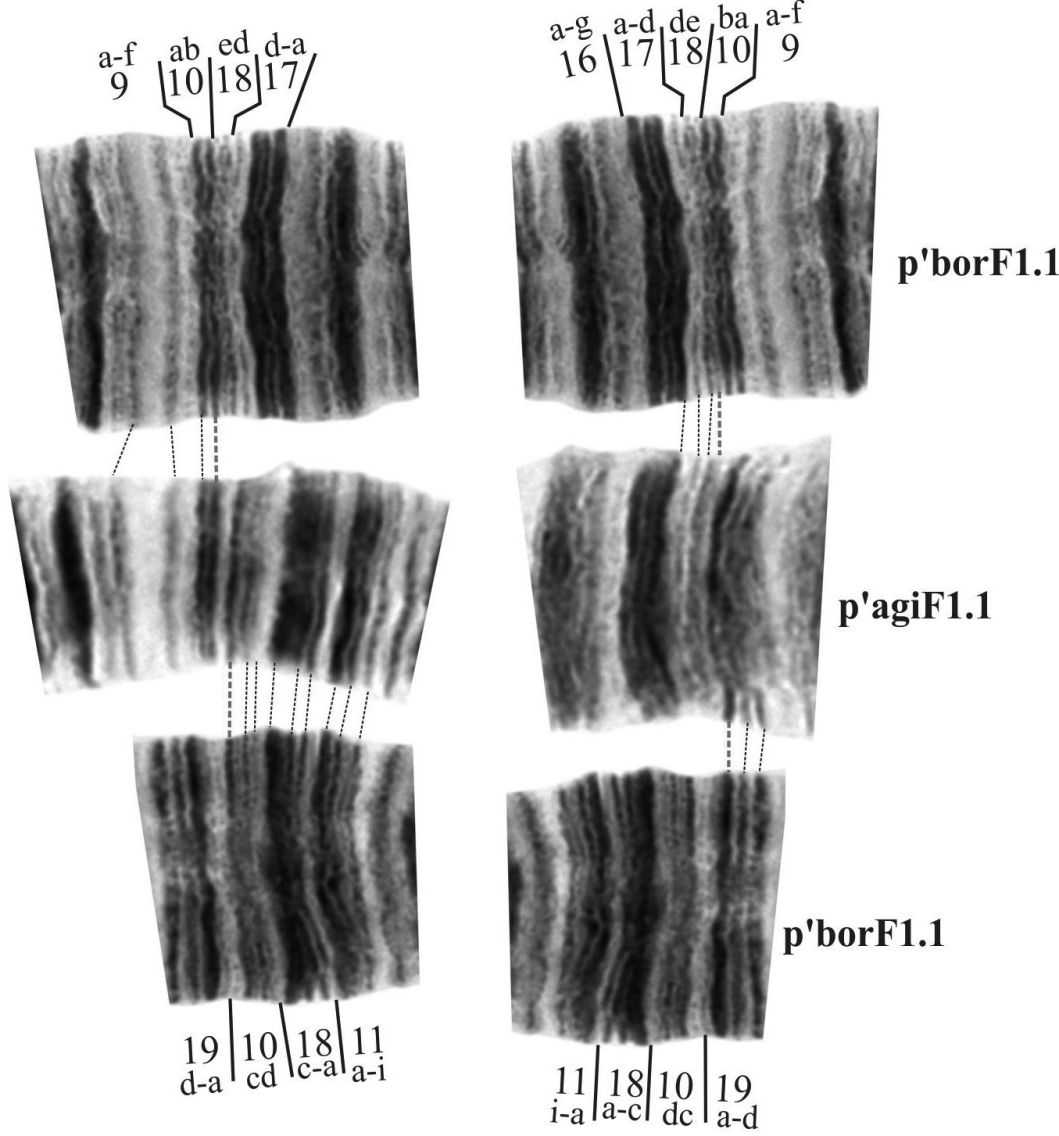
Comparison of regions of inversion breakpoint between banding sequences p’borF1 and p’agiF1.

**Figure 6.**
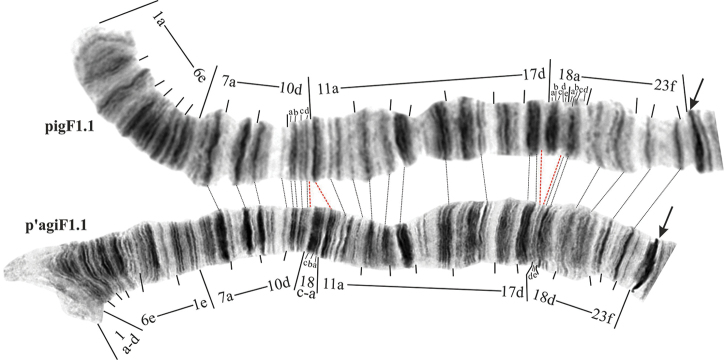
Mapping comparison of banding sequences h’pigF1 and p’agiF1. Centromeric bands are designated by arrows. Individual band in the regions 10, 18 and 19 of h’pigF1 are marked by small letters. Dotted lines connect identical discs in compared banding sequences. Red dotted lines indicate borders of regions, where banding patterns of compared banding sequences are identical.

**Figure 7.**
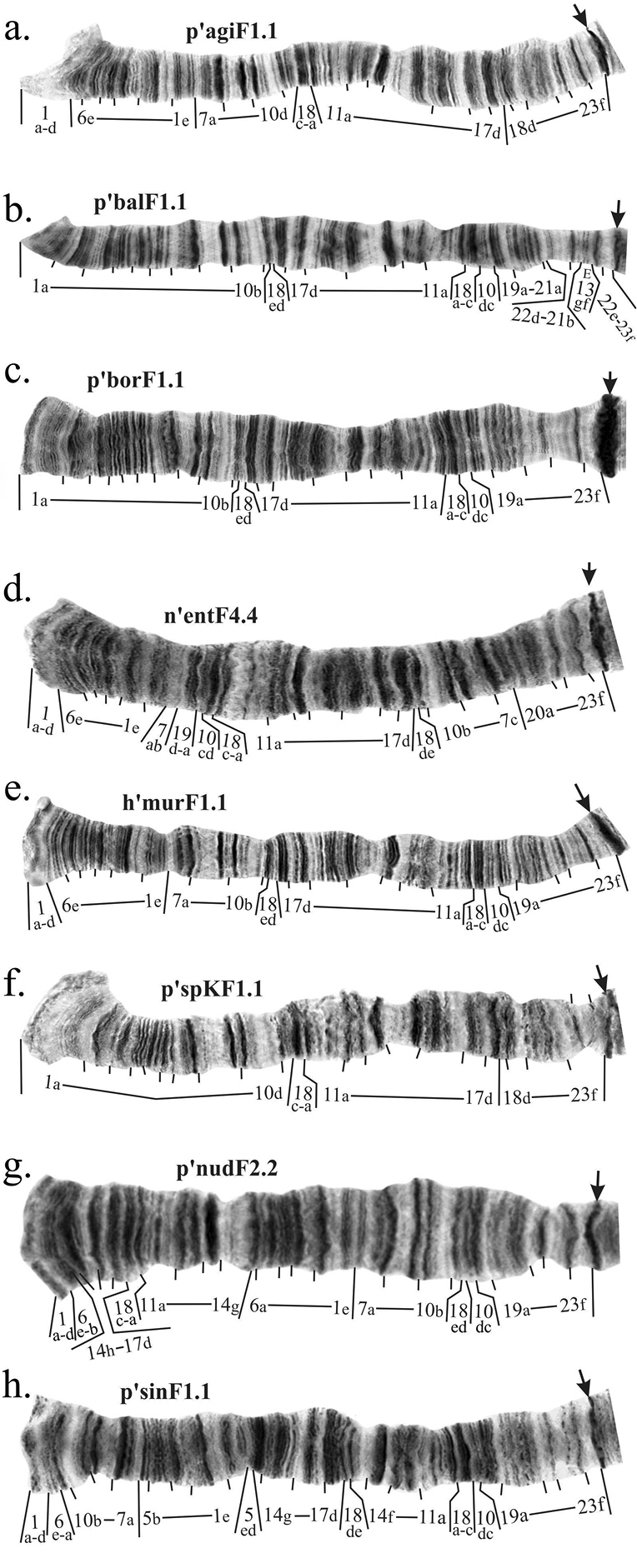
Mapping of banding sequences of *Ch. plumosus* sibling species in arm F after the revision. **a** p’agiF1.1 (identical to p’pluF2, p’ag2F1, p’useF1) **b** p’balF1.1 **c** p’borF1.1 **d** n’entF4.4 **e** h’murF1.1 (identical to h’pluF1, h’bonF1, h’entF1, h’nudF1, h’spJF1) **f** p’spKF1.1 (identical to p’borF2, p’suwF1) **g** p’nudF2.2 **h** p’sinF1.1. Centromeric bands are designated by arrows..

Photos in Figure 5 show the comparison of regions where breakpoints of this inversion occur between banding sequences p’agiF1 and p’borF1 as they had the best banding structure and their mapping could resolve mapping for the rest of banding sequences in the arm. As can be clearly seen, on the left the real inversion breakpoint occurs after band 10b, and on the right – before band 19a. Now it was necessary to determine in which banding sequences region 10 remains whole and in which it breaks and what happens with region 18. To answer these questions it was necessary to compare these banding sequences to h’pigF1. The comparison of p’agiF1 and h’pigF1 is shown on Figure 6. After analysis of arm F from many preparations of *Ch. piger* we concluded that in *Ch. piger* groups of bands 10ab and 10cd have about the same intensity, they are both rather dark and group 10cd is slightly wider than group 10ab. At the same time group 18ed is less dark and defined and often these two bands are so close that it is hard to distinguish them. As can be seen of Figure 6 it is viable to conclude that region 10 stays whole in banding sequence p’agiF1 while region 18 breaks so that bands 18de stay in place before region 19 while 18a-c falls inside the inversion that differentiates it from h’pigF1 and so are transferred to the distal part of the arm near region 10. At the same time region 11a-17d is affected by second inversion in p’agiF1 which results in final banding sequence 1a-d 6e-1e 7a-10d 18c-a 11a-17d 18d-23f (Figs 6, 7a, Table 4). This means that in p’borF1 and, thus, in h’pluF1 and all its homologous and derivatives banding sequences region 10 breaks (Figs 5, 7b-e, g, h, Table 4). It also means that the banding sequence that is closest to h’pigF1 is p’borF2 (and its homologous banding sequences) as it is identical to p’agiF1 in the proximal part of the arm but also have intact banding pattern in region 1a-10d (Fig. 7f, Table 4).

Aside from the general revision that affects all banding sequences in arm F of all species in the group, we have found that arm F of *Ch. balatonicus* required a major revision due to the presence of the pericentric inversion and several banding sequences of different species were in need of breakpoint correction.

### The revision of arm F mapping of *Ch. balatonicus*

As was mentioned above, thorough analysis of the centromeric region of chromosome EF of *Ch. balatonicus* had shown that this arm had undergone complex pericentric inversion that differentiates it from the rest of *Ch. plumosus* group and thus p’balF1 is not identical to p’borF1 as was supposed previously. Mapping of this inversion proved to be very difficult due to the complexity of the rearrangement along with the fact that bands in the pericentromeric region are often weak, not well defined and can be very similar in appearance. The comparison of inversion region between p’balE1, p’balF1, h’nudE1 and h’nudF1 is shown on Figure 4 (photos of h’nudE1 and h’nudF1 were used instead of h’borE1 and p’borF1 as *Ch. nudiventris* has much better structure in the pericentromeric region where the inversion of interest is located). We believe that p’balF1 is a result of three consecutive inversions from p’borF1 so that arm F of *Ch. balatonicus* became longer by addition of bands 13gf from arm E and regions 21 and 22 had rearranged in a complex pattern (Figs 4, 7b, Table 4). 
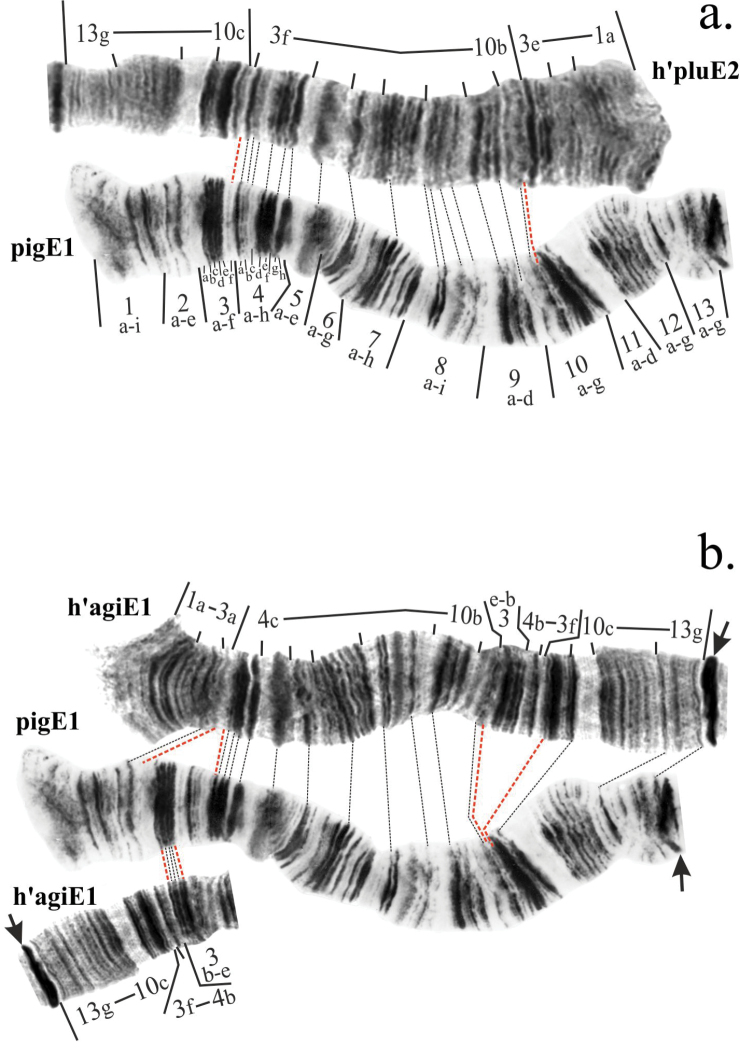


### The revision of arm F mapping of *Ch. entis* and *Ch. nudiventris*

Main banding sequences h’entF1 and h’nudF1 are identical to h’pluF1 (Fig. 2d) so their mapping was changed in accordance with new mapping of h’pluF1. Banding sequence n’entF4 differs from h’entF1 by one simple inversion and we believe that correction should be made for the inversion breakpoints as the inversion divides region 7 (Figs 7d, Table 4). Banding sequence p’nudF2 was previously mapped only in the Maximova system with two inversion steps suggested between it and h’nudF1. However, our analysis has shown that it is much more complex and could be derived from h’nudF1 only by five inversion steps (Figs 2d, 7g, Table 4). What we find very unusual is that so far we found no intermediary banding sequences in the banding sequence pool of *Ch. nudiventris* yet this inversion differs by only four inversion steps from p’pluF2, i.e. it is closer to banding sequence of a sibling species than to main banding sequence of its own species. It is possible that banding sequences identical to p’pluF2 either exists in *Ch. nudiventris* banding sequence pool but was not found yet due to insufficient number of populations studied or existed previously but was lost during speciation. It may also be an evidence that this banding sequence is very old and originated before species speciation of *Ch. nudiventris*.

### The revision of arm F mapping of *Ch. sinicus*

Main banding sequence p’sinF1 differ from h’pluF1 by 3 inversion steps and aside from corrections that follow from changes made to h’pluF1 require a minor revision of inversion breakpoints (Figs 2d, 7h, Table 4).

## Discussion

As was observed in many previous studies, arm E remains the least polymorphic arm of the karyotype in the group. Out of 14 species 10 have identical main banding sequences with another 2 having the same banding sequence as alternative. Only one species – *Ch. balatonicus* – doesn’t share any banding sequences with other species due to the presence of pericentric inversion in the chromosome EF. This species is also the only one that differs from others by more than one inversion step in arm E.

**Table 4.** Mapping of arm F main and alternative banding sequences in *Ch. plumosus* group after the revision.

Designation of banding sequence	Mapping of banding sequence
p’agiF1	=p’pluF2
p’agi2F1	=p’pluF2
p’balF1	1a-10b 18ed 17d-11a 18a-c 10dc 19a-**21a 22d-21b [13gf] 22e-23f** C
p’bonF1	=h’pluF1
p’borF1	1a-**10b 18ed** 17d-11a **18a-c 10dc 19a**-23f C
p’borF2	**1a-10d 18c-a 11a-17d 18d-23f C**
h’entF1	=h’pluF1
n’entF4	1a-d 6e-1e **7ab 19d-a 10cd 18c-a** 11a-17d **18de 10b-7c** 20a-23f C
h’murF1	=h’pluF1
h’nudF1	=h’pluF1
p’nudF2	1a-d 6e-b 14h-17d 18c-a 11a-14g 6a-1e 7a-10b 18ed 10dc 19a-23f C
h’pluF1	1a-d 6e-1e 7a-**10b 18ed** 17d-11a **18a-c 10dc 19a**-23f C
p’pluF2	1a-d 6e-1e 7a-**10d 18c-a** 11a-**17d 18d**-23f C
p’sinF1	1a-d **6e-a 10b**-7a **5b**-1e **5cd 14g**-17d **18de 14f**-11a **18a-c 10dc 19a**-23f C
h’spJF1	=h’entD1
p’spKF1	=p’borF2
p’suwF1	=p’borF2
p’useF1	=p’pluF2

† – parts of the sequences highlighted in bold indicate regions which mapping had been changed as a result of the revision.

The revision in arm F has also mostly provided minor changes in the mapping of inversion breakpoints without affecting phylogenetic relationship of banding sequences inside the group. Aside of the placement of h’balF1 due to the presence of pericentric inversion the only significant change has come from the correction of inversion breakpoint of p’nudF2 which made it related to both h’nudF1 and p’pluF2. In general banding sequences in arm F show a moderate level of divergence comparable with what we observe in arm A, with three species that have species specific main banding sequences and only two species – *Ch. balatonicus* and *Ch. sinicus* – that don’t share any banding sequence with other species.

Considering the level of banding sequences divergence in both arms it can be stated that chromosome EF is the least divergent among the three big chromosomes of *Chironomus*.

At the same time the revision has shown the phylogenetic relationship of banding sequences of *Ch. plumosus* group sibling species and the rest of the genus are different from what was assumed previously as the closest to h’pigE1 and h’pigF1 are h’pluE2 and p’borF2. As both arm E and F are low polymorphic in the genus, many species share the same banding sequences. As was mentioned previously, h’pluE1 and p’borF1 were believed to be identical to banding sequences of many other species as their banding patterns are considered basic for the genus *Chironomus* (Keyl 1962, Wülker 1980, Kiknadze et al. 2004a, 2016). Because of the revision presented for these banding sequences in this paper we are now facing two possibilities. First, it is possible that they are indeed identical to banding sequences of other species in the genus, in which case a revision of banding sequences in arms E and F required for the most species of the genus. Second, there is a possibility that these banding sequences are actually different from the basic banding sequences, in which case only mapping of banding sequences in the *Ch. plumosus* group is affected. To answer the question of which assumption is correct it is necessary to compare these banding sequences to basic banding sequences found in other species of the genus *Chironomus*.
